# Cellular senescence primes liver fibrosis regression through Notch‐EZH2

**DOI:** 10.1002/mco2.346

**Published:** 2023-08-21

**Authors:** Ping Song, Juan‐Li Duan, Jian Ding, Jing‐Jing Liu, Zhi‐Qiang Fang, Hao Xu, Zhi‐Wen Li, Wei Du, Ming Xu, Yu‐Wei Ling, Fei He, Kai‐Shan Tao, Lin Wang

**Affiliations:** ^1^ Department of Hepatobiliary Surgery Xi‐Jing Hospital Fourth Military Medical University Xi'an China

**Keywords:** EZH2, liver fibrosis regression, macrophage, Notch, senescence

## Abstract

Cellular senescence plays a pivotal role in wound healing. At the initiation of liver fibrosis regression, accumulated senescent cells were detected and genes of senescence were upregulated. Flow cytometry combined with single‐cell RNA sequencing analyses revealed that most of senescent cells were liver nonparenchymal cells. Removing senescent cells by dasatinib and quercetin (DQ), alleviated hepatic cellular senescence, impeded fibrosis regression, and disrupted liver sinusoids. Clearance of senescent cells not only decreased senescent macrophages but also shrank the proportion of anti‐inflammatory M2 macrophages through apoptotic pathway. Subsequently, macrophages were depleted by clodronate, which diminished hepatic senescent cells and impaired fibrosis regression. Mechanistically, the change of the epigenetic regulator enhancer of zeste homolog2 (EZH2) accompanied with the emergence of hepatic senescent cells while liver fibrosis regressed. Blocking EZH2 signaling by EPZ6438 reduced hepatic senescent cells and macrophages, decelerating liver fibrosis regression. Moreover, the promoter region of EZH2 was transcriptionally suppressed by Notch‐Hes1 (hairy and enhancer of split 1) signaling. Disruption of Notch in macrophages using Lyz2 (lysozyme 2) ^Cre^‐RBP‐J (recombination signal binding protein Jκ) ^f/f^ transgenic mice, enhanced hepatic cellular senescence, and facilitated fibrosis regression by upregulating EZH2 and blocking EZH2 abrogated the above effects caused by Notch deficiency. Ultimately, adopting Notch inhibitor Ly3039478 or exosome‐mediated RBP‐J decoy oligodeoxynucleotides accelerated liver fibrosis regression by augmenting hepatic cellular senescence.

## Graphical abstract

Senescent liver nonparenchymal cells accumulated at the beginning of liver fibrosis regression. Clearance of these senescent cells disrupted fibrosis regression through limiting anti‐inflammatory macrophages. The epigenetic regulator EZH2, which was transcriptionally suppressed by Notch‐Hes1 signaling, was proven to be the molecular determinant of regression‐related senescence. Blocking Notch‐Hes1 signaling by Notch inhibitor or targeted delivery of exosome‐mediated Notch/RBP‐J decoy facilitated fibrosis regression by enhancing EZH2‐regulated senescence.

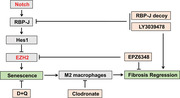

## 1 INTRODUCTION

Chronic liver injuries lead to liver fibrosis, by activating hepatic extracellular matrix‐derived myofibroblasts.^1^ When the causative injuries are removed, clinical and experimental liver fibrosis may regress.^1^, ^2^ Hepatocytes, hepatic stellate cells (HSCs), liver sinusoidal endothelial cells (LSECs), and immune cells, particularly macrophages, cooperate to initiate the regression of liver fibrosis.^3^, ^4^, ^5^ Accumulated investigations focus mainly on the progression of liver fibrosis; however, fibrosis regression is relatively understudied.^6^ To investigate the underlying mechanisms of regression, a new therapeutic strategy may be established to treat liver fibrosis.^7^

Cellular senescence is a permanent state of cell cycle arrest.^8^ Pathways of P16^Ink4a^ and P53/P21, which are important for irreversible growth arrest, have been widely recognized as regulators for cellular senescence.^9^ Senescent cells not only originate from age‐related diseases,^10^ but also emerge in tumor suppression,^11^ tissue remodeling,^12^ wound healing,^13^ and protection against organ fibrosis,^14^ The coordinated induction of senescence benefits fibrotic scar removal through senescence‐associated secretory phenotype (SASP).^15^ Hepatic stellate cell‐derived senescence limited fibrogenesis^15^ and depletion of p16^High^ hepatic senescent cells accelerated fibrosis.^16^ However, misregulated senescence deteriorated organ remodeling. Application of senolytics to remove senescent cells, improved liver regeneration^17^ and ameliorated steatohepatitis.^18^ Recently, our group reported that shear stress‐induced endothelial cellular senescence impaired liver regeneration following partial hepatectomy.^19^ Thus, the role of senescent cells in response to liver injury is still controversial.

EZH2 is a histone H3K27me3 methyltransferase.^20^ EZH2 usually functions as an epigenetic regulator of cell cycle,^21^ autophagy,^22^ apoptosis,^23^ and DNA damage repair.^24^ Numerous studies have identified the regulatory role of EZH2 in many diseases,^25^ including cancer.^26^, ^27^ As the change of the expression of epigenetic genes is associated with senescence‐induced phenotype,^28^ the importance of EZH2 in driving cellular senescence attracts our interests. P21 is known as a landmark gene of senescence.^29^ EZH2 can directly bind to the promoter region of P21 and facilitate H3K27me3 modification to orchestrate senescence.^30^ Although our previous findings proved that shear stress‐induced cellular senescence was accompanied with the upregulation of EZH2 at the late phase of liver regeneration,^19^ the detailed regulatory role of EZH2 in manipulating liver injury‐induced senescence was not fully elucidated. Therefore, we selected EZH2 as a key molecule in the regulation of senescence. In this study, we found that EZH2 was transcriptionally suppressed by Hes1, a direct downstream gene of Notch signaling.^31^ Once RBP‐J, the main transcriptional mediator of Notch,^32^ was disrupted, the effect of EZH2‐regulated senescence could be reversed. The potential link between Notch and EZH2 unveils a brand new mechanism in the regulation of cellular senescence.

In this study, we found that EZH2‐regulated senescent cells accumulated while liver fibrosis regression began. Disruption of the epigenetic regulation of EZH2 removed anti‐inflammatory senescent cells and impaired fibrosis regression. As the transcription of EZH2 was impeded by Notch‐Hes1 signaling, blocking Notch facilitated fibrosis regression by strengthening EZH2‐regulated senescence, providing a promising therapeutic approach for the management of liver fibrosis.

## 2 RESULTS

### 2.1 Accumulated senescent cells emerge at the beginning of liver fibrosis regression

**FIGURE 1:**
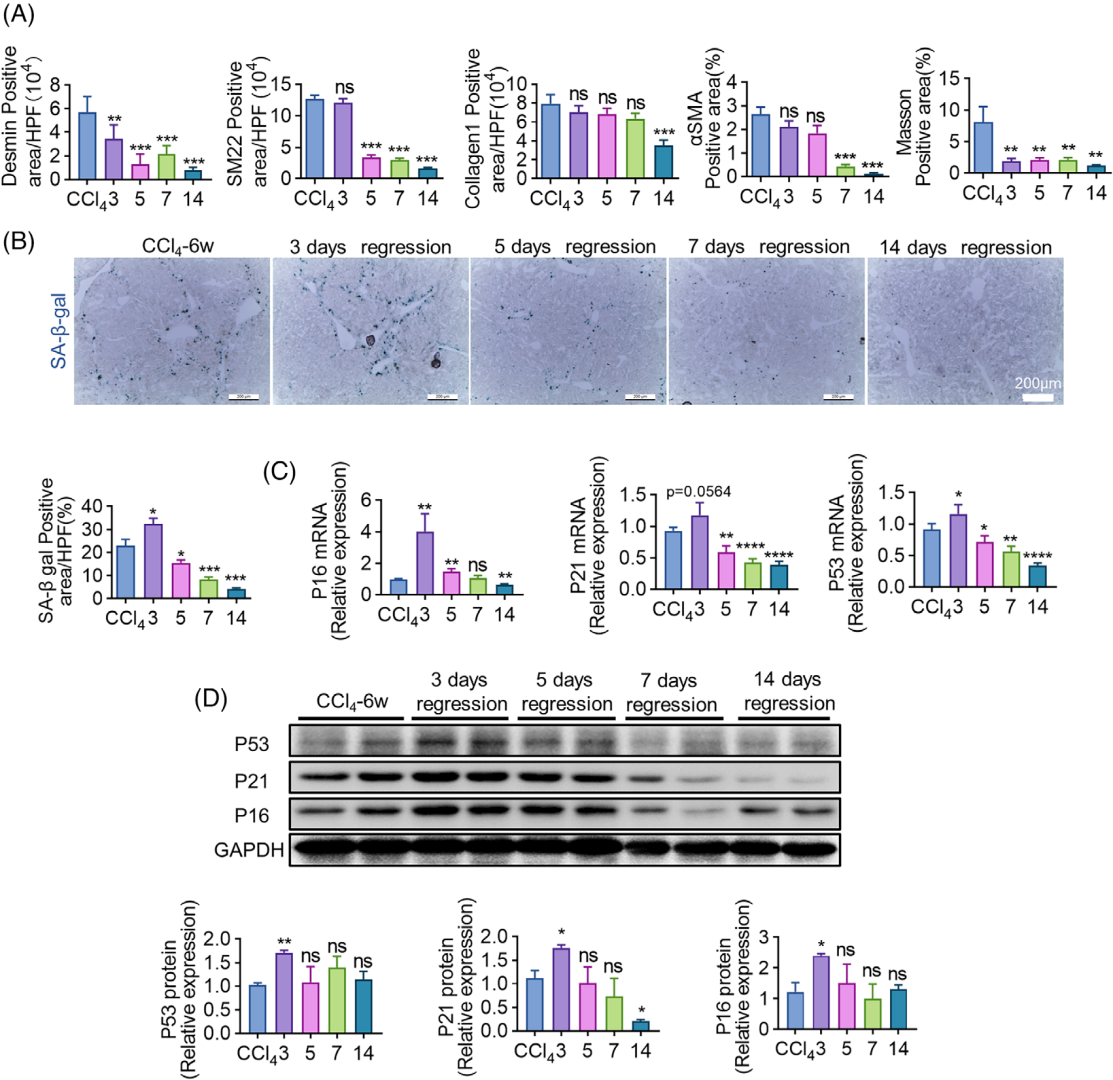
The emergence of accumulated senescent cells during fibrosis regression. (A) The quantification of positive areas of IF (Desmin, SM22, Collagen1) and IHC (αSMA, Masson) staining of livers on days 3, 5, 7, and day 14 of regression. Mice sacrificed immediately after the 12th CCl_4_ injection served as control. (B) Frozen sections of livers were stained with a Senescence β‐galactosidase Staining Kit (SA‐β‐gal Kit). Positive areas of SA‐β‐gal were quantitatively compared. (C) Relative mRNA expressions of senescence‐related molecules including P16, P21, and P53 in isolated macrophages were determined using RT‐qPCR during the regression of liver fibrosis; β‐actin gene was used as an internal reference. (D) WB analyses of P16, P21, and P53 expression of isolated macrophages at various time points of regression; GAPDH served as an internal control. Bars = means ± SD; *n* = 2–4; *p < 0.05, **p < 0.01, ***p < 0.001, ****p < 0.0001; ns, not significant.

Liver fibrosis was induced by 6‐week CCl_4_ (carbon tetrachloride) injection.^33^ To evaluate fibrosis regression, mice were sacrificed and analyzed on 1, 2, 3, 4, 5, 7, or 14 days following the removal of CCl_4_. An increase of liver/body weight ratio was found on day 3 of regression (Figure S1A). Serum ALT and AST levels peaked on day 1 and declined subsequently on day 3 (Figure S1B), which was consistent with the change of Masson staining (Figure S1C,D). As shown by the immunofluorescent (IF) and IHC (immunohistochemistry) staining (Figure 1A and Figure S1E), fibrosis‐associated markers like Desmin and Masson significantly decreased on day 3, while SM22 (smooth muscle 22) and αSMA (alpha smooth muscle actin) declined on days 5 and 7, respectively, after CCl_4_ withdrawal, confirming the resolution of liver fibrosis. SA‐β‐gal (senescence‐associated β‐galactosidase) is the most commonly utilized marker for identifying senescent cells.^34^ Interestingly, the maximum level of SA‐β‐gal staining was observed 3 days after CCl_4_ withdrawal (Figure 1B). Additionally, RT‐qPCR (real‐time quantitative PCR) analyses of primary macrophages revealed an upregulation in terms of gene expression related to senescence, including P16, P21, and P53 on day 3 of regression (Figure 1C). Western blotting (WB) quantification showed that protein levels of P16, P21, and P53 peaked on day 3 of regression (Figure 1D). These data collectively proved that cellular senescence emerged at the beginning of fibrosis regression.

### 2.2 Senescent cells predominantly originate from liver nonparenchymal cells

**FIGURE 2:**
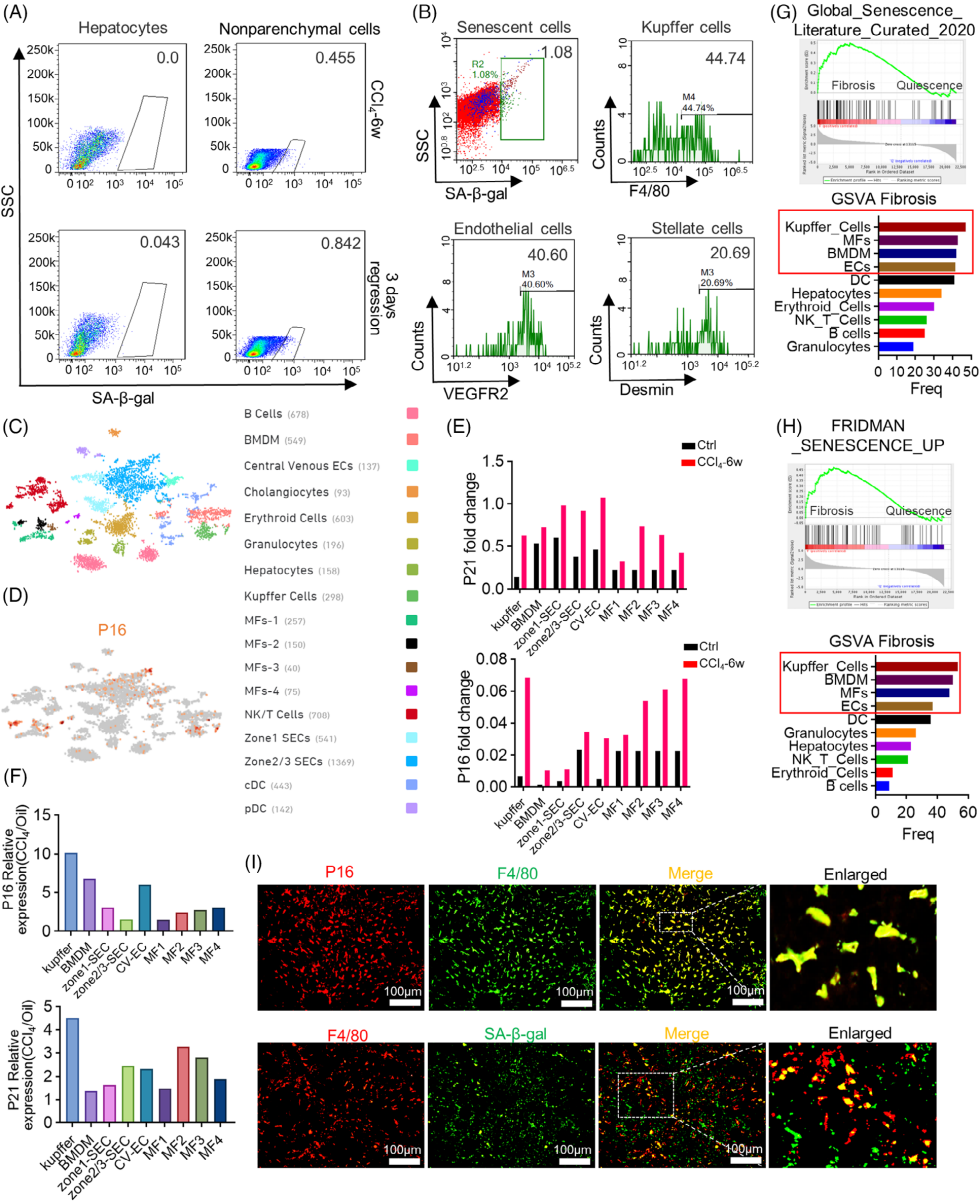
Identification of the origin of senescent cells. (A) Single‐cell suspensions collected from hepatocytes or liver NPCs were analyzed by FCM 3 days after the withdrawal of CCl_4_; *n* = 1. (B) FCM analyses showed the proportion of KCs (F4/80), LSECs (VEGFR2), and HSCs (desmin) in senescent NPCs (SA‐β‐gal) on regression day 3. (C) tSNE (t‐distributed stochastic neighboring embedding) clustering representation of the distinct cell types of liver NPCs following CCl_4_ toxicity. (D) Gene expression of P16 was visualized by tSNE feature plots. (E and F) Fold change in expression of P16 and P21 observed in individual cell types by scRNA‐seq. (G and H) GSEA analyses with Global Senescence Literature Curated 2020 or Fridman Senescence UP in mice with CCl_4_ removal and control mice. GSVA enrichment score was performed for the above two senescence‐related gene sets of both experimental and control mice, bar graphs represented the frequency basis of positive cells in various NPC types by GSVA score. (I) IF co‐staining of P16 (red)/F4/80 (green) or SA‐β‐gal (green)/f4/80 (red) of livers on regression day 3. Scale bar: 100 μm. Bars = means ± SD; *n* = 3.

Next, we try to clarify the origin of the senescent cells. Flow cytometry (FCM) demonstrated that a significant increase of SA‐β‐gal^+^ senescent cells on regression day 3 could be detected in liver nonparenchymal cells (NPCs) (from 0.455% to 0.842%) but not hepatocytes (Figure 2A). Further analyses revealed that 44.74% of senescent cells were F4/80^+^ KCs (Kupffer cells), 40.60% were VEGFR2^+^ LSECs, and 20.69% were Desmin^+^ HSCs (Figure 2B). These FCM data proved that senescent cells dominantly originated from liver NPCs, especially KCs and LSECs. To confirm these findings, scRNA‐seq (single‐cell RNA sequence) was manipulated in mice with fibrosis regression. The distribution of different hepatic cells and P16^+^ senescent cells is shown in Figure 2C,D. The gene set enrichment analysis (GSEA) and gene set variation analysis (GSVA) identified that senescence marker genes, which enriched in livers with fibrosis regression, mostly expressed in KCs, BMDM (bone marrow‐derived macrophages), MFs (myofibroblasts), and LSECs (Figure 2G,H). We then compared the expression of marker genes of senescence between control and experimental mice by scRNA‐seq. The upregulation of P16 and P21 expression was observed in hepatic cells of mice with fibrosis regression (Figure 2E). However, the biggest alteration of P16 and P21 could be found in KCs (Figure 2F). Additionally, IF staining confirmed the co‐stain of macrophages (F4/80^+^) and senescent cells (P16^+^ or SA‐β‐gal^+^) on regression day 3 (Figure 2I). Collectively, these findings preliminarily implied that hepatic macrophages devoted major contribution to the emergence of cellular senescence.

### 2.3 Depletion of senescent cells inhibits liver fibrosis regression and disrupts hepatic sinusoids

**FIGURE 3:**
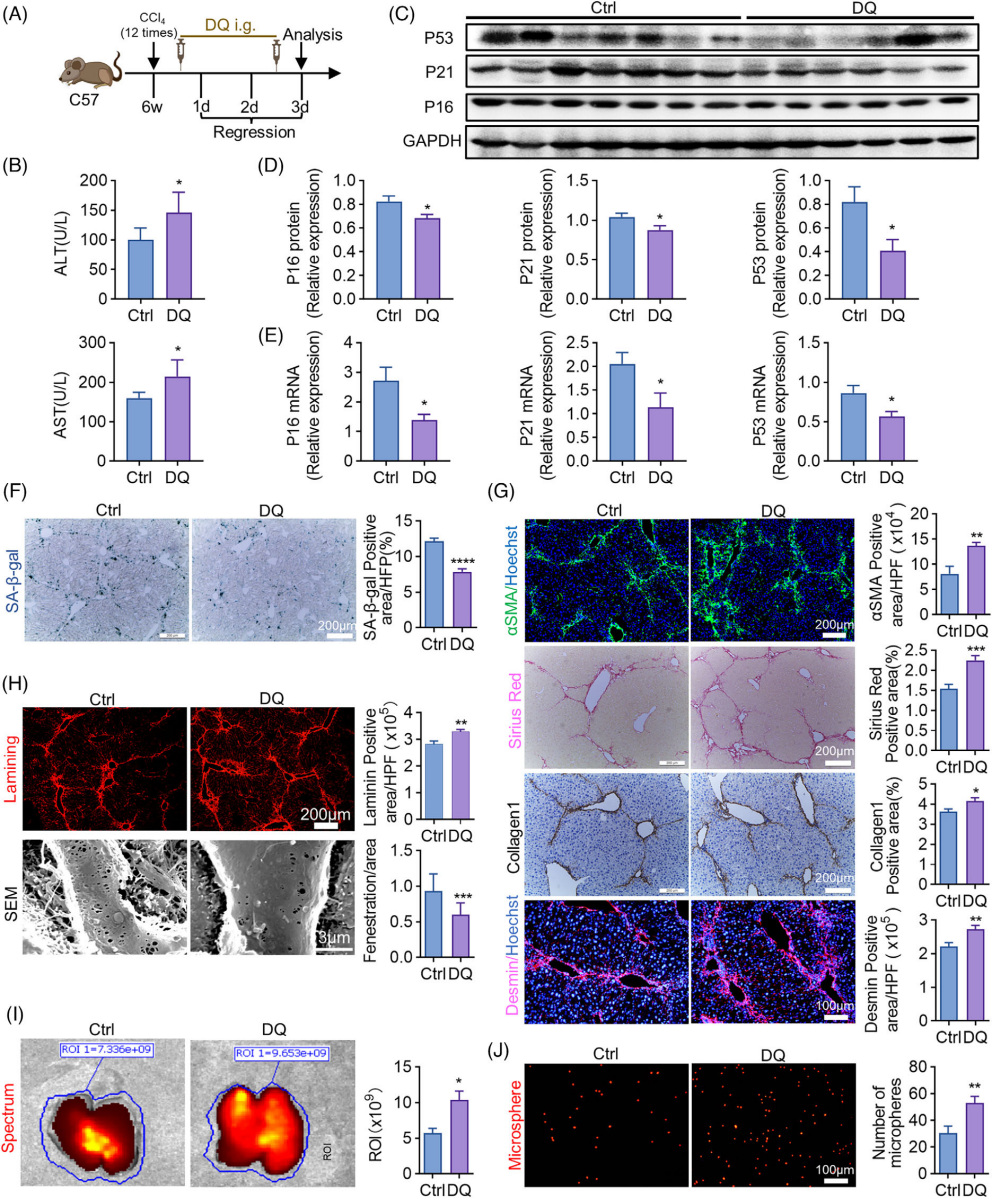
Removing senescent cells inhibits liver fibrosis regression. (A) The strategy of DQ (dasatinib and quercetin) administration during the regression of liver fibrosis. All the experimental mice were sacrificed and analyzed on regression day 3. The cartoon was created by Photoshop. i.g. means oral gavage. (B) Detection of serum ALT, AST in DQ‐treated and control mice. (C) WB analyses of P16, P21, and P53 expressions in KCs of DQ‐treated and control mice; GAPDH served as an internal reference. (D) The quantification of (C). (E) The mRNA levels of P16, P21, and P53 were detected by RT‐qPCR in DQ‐treated and control mice; β‐actin was used as an internal reference. (F) Liver sections collected from DQ‐treated and control mice were stained by SA‐β‐gal staining kit. SA‐β‐gal‐positive cells were quantitatively compared. Scale bar: 200 μm. (G) IF (αSMA, Desmin) and IHC (Sirius red, Collagen1) staining of DQ‐treated and control mice. Positive areas were quantified and compared. Scale bar: 200 μm (αSMA, Sirius red, Collagen1), scale bar: 100 μm (desmin). (H) Staining of Laminin in DQ‐treated and control mice. Positive areas were quantitatively compared. Scale bar: 200 μm. Sinusoidal fenestrae were examined and quantitatively compared by SEM in DQ‐treated and control mice. Scale bar: 3 μm. (I and J) Red fluorescent microspheres injected into the spleen were subsequently observed in the liver. The fluorescence intensity was compared in (I) and the microspheres were quantified in (J). Scale bar: 100 μm. Bars = means ± SD; *n* = 6; *p < 0.05, **p < 0.01, ****p < 0.0001.

Subsequently, DQ which is one of the classical senolytics,^35^ was intragastric administration on regression day 0.5 and day 2.5 to remove senescent cells. Mice were analyzed on day 3 as previously (Figure 3A). After DQ administration, both serum ALT (alanine aminotransferase) and AST (aspartate aminotransferase) levels increased (Figure 3B), implying the liver function deteriorated after senescent cell depletion. WB (Figure 3C,D) and RT‐qPCR (Figure 3E) analyses confirmed the downregulation of senescence markers, including P16, P21, and P53. SA‐β‐gal staining also reflected the successful removal of senescent cells (Figure 3F). Next, we observed the change of liver fibrosis. Apparently, αSMA, Sirius red, collagen1, and desmin staining all illustrated that liver fibrosis was aggravated by DQ (Figure 3G), indicating removing senescent cells reversed fibrosis regression.

Besides, enhanced laminin stain and reduced LSEC fenestration, which were determined by IF staining and SEM (scanning electronic microscopy), respectively, confirmed the capillarization of liver sinusoids (Figure 3H). To assess alterations in blood flow within sinusoids, fluorescent polystyrene microspheres were injected into the spleen and subsequently detected within the liver. Bioluminescence imaging analysis showed enhanced liver fluorescence after DQ treatment (Figure 3I). Furthermore, increased fluorescent microspheres were detected in livers of mice with DQ administration (Figure 3J). These results implied that liver sinusoids were occluded once senescent cells were removed.

### 2.4 Removing senescent cells diminishes anti‐inflammatory macrophages through apoptotic pathway

**FIGURE 4:**
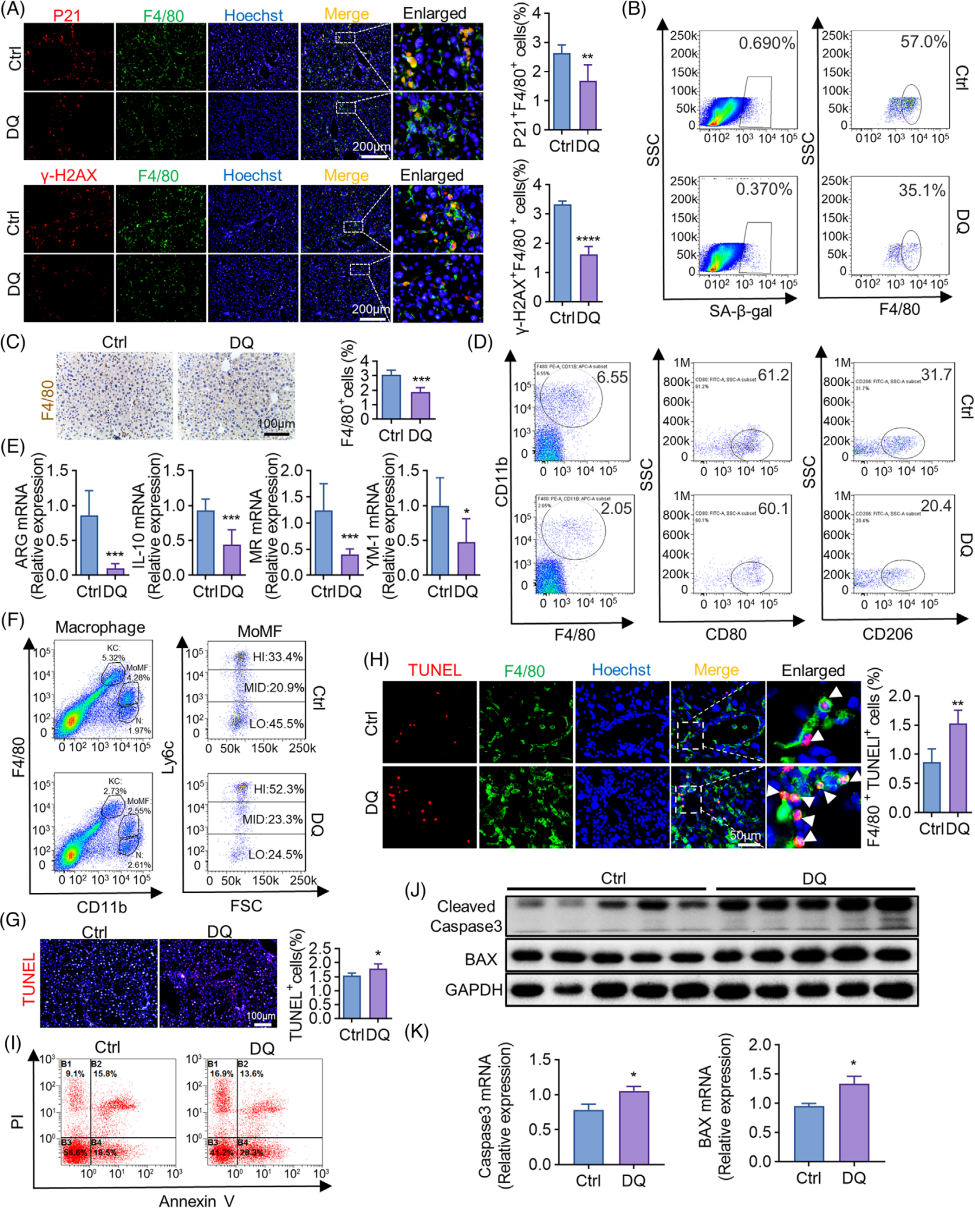
Clearance of senescent cells diminishes M2 macrophages. All the experimental mice were sacrificed and analyzed on regression day 3. (A) IF co‐staining of F4/80 (green) and P21 (red) or r‐H2AX (red)/f4/80 (green) in livers of DQ‐treated and control mice. Double positive cells were quantitatively compared. Scale bar: 200 μm. (B) The proportion of SA‐β‐gal^+^ or F4/80^+^ cells in liver NPCs of DQ‐treated and control mice was analyzed by FCM. (C) Liver sections collected from DQ‐treated and control mice were analyzed using anti‐F4/80 IHC. F4/80‐positive cells were quantitatively compared. Scale bar: 100 μm. (D) Isolated liver NPCs from DQ‐treated or control mice were analyzed by FCM to detect the change of polarization (M1:CD80; M2:C206). (E) KCs were magnetically isolated from DQ‐treated and control mice. Molecular markers of M2 macrophages were determined by RT‐qPCR, with β‐actin as an internal control. (F) Isolated liver NPCs from DQ‐treated and control mice were analyzed by FCM to detect F4/80^+^CD11b^+^Ly6C^hi^ or F480^+^CD11b^+^Ly6C^lo^ proportions. MoMF, monocyte‐derived macrophages; KC, Kupffer cells; *N*, neutrophils; HI, high; MID, middle; LO, low. (G and I) TUNEL staining and FCM were used to detect apoptotic liver cells in DQ‐treated and control mice. TUNEL^+^ cells were quantitatively compared (G). Scale bar: 100 μm. (H) IF co‐staining of TUNEL (red) and F4/80 (green) in livers of DQ‐treated and control mice. Double positive cells were quantitatively compared. Scale bar: 50 μm. (J) Protein levels of cleaved caspase3 and BAX in KCs isolated from DQ‐treated and control mice were determined by WB; GAPDH served as an internal control. (K) RT‐qPCR analyses of cleaved caspase3 and BAX in KCs isolated from DQ‐treated and control mice. Bars = means ± SD; *n* = 3–6; *p < 0.05, **p < 0.01, ***p < 0.001, ****p < 0.0001.

We then try to clarify how senescent cells facilitate liver fibrosis regression. According to IF staining, P21^+^ or γ‐H2AX^+^ senescent macrophages were greatly reduced by DQ on regression day 3 (Figure 4A). FCM analyses also confirmed the decrease of macrophage senescence (from 57.0% to 35.1%) after DQ administration (Figure 4B), which proved that hepatic macrophages attributed to the emergence of senescence. Besides, total macrophages of livers treated with DQ decreased significantly (Figure 4C). As we know, macrophages usually stimulate liver fibrosis in different models.^36^ However, our findings showed that depletion of senescent cells decreased hepatic macrophages and impedes fibrosis regression. To illustrate this contradiction, FCM analyses were performed in hepatic macrophages between control and DQ‐treated mice. As shown in Figure 4D, DQ removed F4/80^+^CD11b^+^ hepatic macrophages majorly through reducing M2 (from 31.7% to 20.4%), but not M1 macrophages. Marker genes of M2 macrophages like ARG (arginase), IL‐10 (interleukin‐10), MR (mannose receptor c‐type 1), and YM‐1 (chitinase‐like 3) were all downregulated by DQ, which were determined by RT‐qPCR (Figure 4E). As M2 macrophages normally take anti‐inflammatory effects,^37^ we then evaluated the change of anti‐inflammatory macrophages following DQ administration. As expected, DQ significantly lowered the percentage of Ly6c^Low^ macrophages (from 45.5% to 24.5%) (Figure 4F), which have been widely accepted as anti‐inflammatory macrophages.^38^ Thus, we may conclude that most of senescent macrophages induced by fibrosis regression were anti‐inflammatory macrophages.

We then explored the involved mechanism of the reduction of anti‐inflammatory macrophages caused by DQ. Apoptotic liver cells increased after DQ injection, which was determined by TUNEL (terminal deoxynucleotidyl transferase‐mediated dUTP nick‐end labeling) staining (Figure 4G). Besides, TUNEL and F4/80 co‐stain (Figure 4H) and anti‐Annexin V FCM analyses (Figure 4I) both revealed that apoptotic liver macrophages significantly increased once senescent cells were removed. Mechanistically, apoptosis‐associated pathways, such as cleaved caspase 3 and BAX were upregulated by DQ in both protein (Figure 4J) and mRNA levels (Figure 4K), suggesting DQ diminished anti‐inflammatory macrophages through apoptotic pathways. In summary, DQ prevented the fibrosis regression by reducing the anti‐inflammatory macrophages through apoptotic pathways.

### 2.5 Clearance of macrophages alleviates cellular senescence and impedes liver fibrosis regression

**FIGURE 5:**
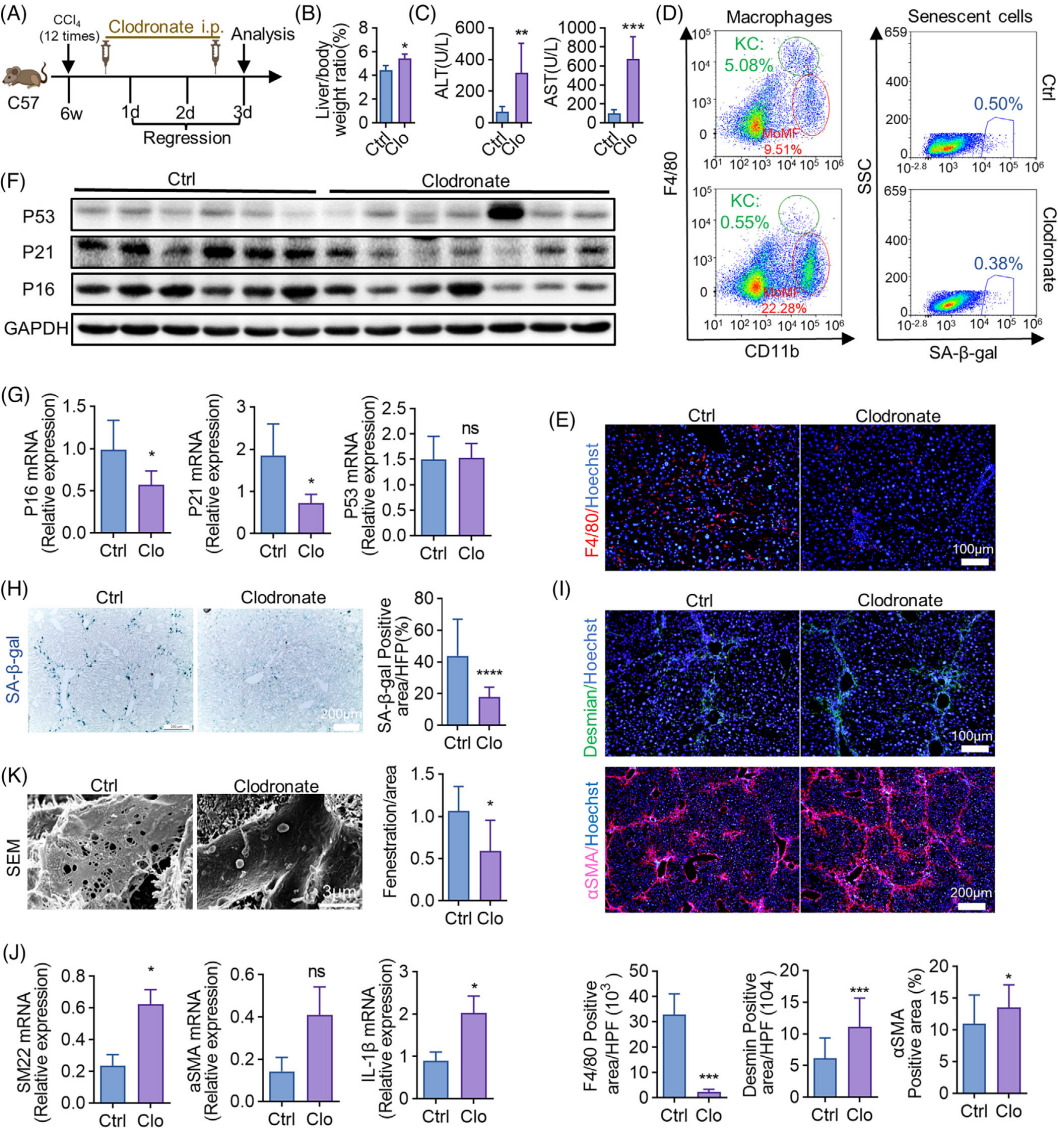
Depletion of macrophages alleviates cellular senescence and impedes liver fibrosis regression. (A) The strategy of clodronate liposome injection. All the experimental mice were sacrificed and analyzed on regression day 3. i.p. = intraperitoneal injection. (B) Liver to bodyweight ratios were measured in clodronate liposome‐treated (Clo) and control mice. (C) Detection of serum ALT, AST levels in clodronate‐treated and control mice. (D) The proportion of SA‐β‐gal^+^ and F4/80^+^ CD11b^+^ in liver NPCs of clodronate‐treated and control mice was analyzed by FCM. (E) Anti‐F4/80 IF staining of livers collected from clodronate‐treated and control mice. F4/80‐positive cells were quantitatively compared. Scale bar: 100 μm. (F) Protein levels of P16, P21, and P53 in livers of clodronate‐treated and control mice were determined by WB; GAPDH served as an internal control. (G) The mRNA levels of P16, P21, and P53 were detected by RT‐qPCR in clodronate‐treated and control mice. (H) Staining of SA‐β‐gal. SA‐β‐gal‐positive cells were quantitatively compared. Scale bar: 200 μm. (I) IF staining and quantification of desmin and αSMA in livers of clodronate‐treated and control mice. Scale bar: 100 μm (desmin), scale bar: 200 μm (αSMA). (J) RT‐qPCR analyses of mRNA expressions of SM22, αSMA, and IL‐1β. (K) SEM analysis of liver sections collected from clodronate‐treated and control mice. Sinusoidal fenestrae were quantitatively compared. Scale bar: 3 μm. Bars = means ± SD; *n* = 4–7; *p < 0.05, **p < 0.01, ***p < 0.001; ns, not significant.

To confirm that senescent cells initiate fibrosis regression through anti‐inflammatory macrophages, we then removed macrophages to evaluate the change of regression. Clodronate injected Intraperitoneally (IP) on regression day 0.5 and day 2.5 (Figure 5A), increased the liver/body weight ratio of mice analyzed on day 3 (Figure 5B). Serum AST and ALT levels were elevated in clodronate‐treated group (Figure 5C). IF staining of F4/80 proved the successful clearance of hepatic macrophages (Figure 5E). FCM analyses further confirmed the decrease of KCs (from 5.08% to 0.55%) and senescent macrophages (from 0.50% to 0.38%) in livers of clodronate‐treated mice (Figure 5D). Importantly, the expression of marker genes of senescence, such as P16 and P21, were downregulated by clodronate in both protein (Figure 5F) and mRNA levels (Figure 5G). SA‐β‐gal staining also showed that removing macrophages diminished hepatic cellular senescence (Figure 5H). Next, Desmin and αSMA IF staining performed in livers of control and clodronate‐treated mice showed that depletion of macrophages impeded fibrosis regression (Figure 5I). The expression of fibrosis‐related genes like SM22, αSMA, and IL‐1β (interleukin‐1β) was downregulated by clodronate as well, which was determined by RT‐qPCR (Figure 5J). Besides, reduced LSEC fenestration was observed in mice with clodronate administration (Figure 5K). These data implied that most of the macrophages removed by clodronate exerted anti‐inflammatory effects during the process of regression.

### 2.6 The epigenetic regulator EZH2 impels cellular senescence and promotes liver fibrosis regression

**FIGURE 6:**
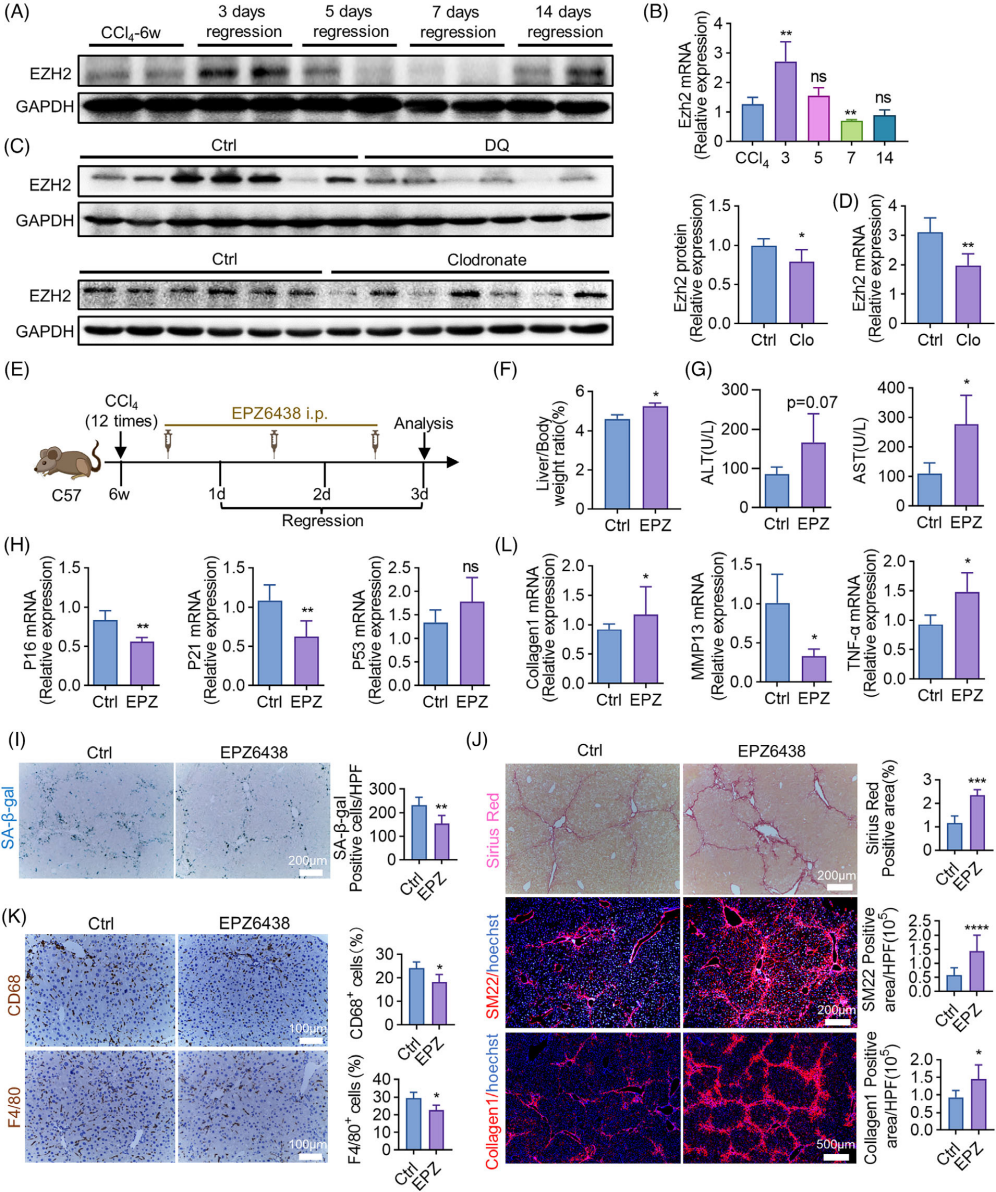
EZH2 promotes cellular senescence and liver fibrosis regression. (A and B) Protein (A) and mRNA (B) levels of EZH2 in isolated macrophages at various time points during liver fibrosis regression; GAPDH and β‐actin served as internal controls. (C and D) Protein (C) and mRNA (D) levels of EZH2 in livers of DQ or clodronate‐treated and control mice on regression day 3. GAPDH and β‐actin served as internal controls. (E) The strategy of EPZ6438 administration. All the experimental mice were sacrificed and analyzed on regression day 3. (F) Liver to body weight ratios were detected in EPZ6438‐treated (EPZ) and control mice. (G) Detection of serum ALT, AST. (H) The mRNA levels of P16, P21, and P53 were detected by RT‐qPCR in EPZ6438‐treated and control mice. (I) Staining of SA‐β‐gal. SA‐β‐gal‐positive cells were quantitatively compared. Scale bar: 200 μm. (J) Sirius red and IF staining (SM22, Collagen1) of livers of EPZ6438‐treated and control mice. Positive areas were quantified and compared. Scale bar: 200 μm (Sirius red, SM22), scale bar: 500 μm (Collagen1). (K) Liver sections obtained from the EPZ6438‐treated or control group were analyzed using immunohistochemical staining with anti‐CD68 and anti‐F4/80. Scale bar: 100 μm. (L) RT‐qPCR analyses of Collagen1, MMP13 (matrix metallopeptidase 13), and TNF‐α (tumor necrosis factor α) in livers of EPZ6438‐treated and control mice. Bars = means ± SD; *n* = 5; *p < 0.05, **p < 0.01, ***p < 0.001, ****p < 0.0001; ns, not significant.

EZH2, an epigenetic modulator, has been regarded as a driver of cellular senescence.^19^ To illustrate the underlying mechanism, the expression of EZH2 was measured during the process of fibrosis regression. In consistent with the change of senescence (Figure 1B), the peaked expression of EZH2 occurred on day 3 as well, which was determined by WB (Figure 6A) and RT‐qPCR (Figure 6B). We then evaluated EZH2 expression in livers with senescent cells or macrophages depletion. DQ and clodronate lowered EZH2 protein level (Figure 6C), and clodronate reduced the mRNA expression of EZH2 (Figure 6D), implying the possibility that DQ and clodronate inhibited senescence and regression through EZH2. To prove this hypothesis, EPZ6438, an inhibitor of EZH2, was administrated three doses during regression (Figure 6E). EPZ6438 increased relative liver weight (Figure 6F) and deteriorated liver function (Figure 6G). Besides, blocking EZH2 by EPZ6438 alleviated cellular senescence (Figure 6H,I), impaired fibrosis regression (Figure 6J,L), and reduced hepatic macrophages (Figure 6K). These findings collectively proved that EZH2‐regulated senescence advanced liver fibrosis regression.

### 2.7 Notch‐Hes1 signaling impedes senescence‐driven fibrosis regression by suppressing the transcription of EZH2

**FIGURE 7:**
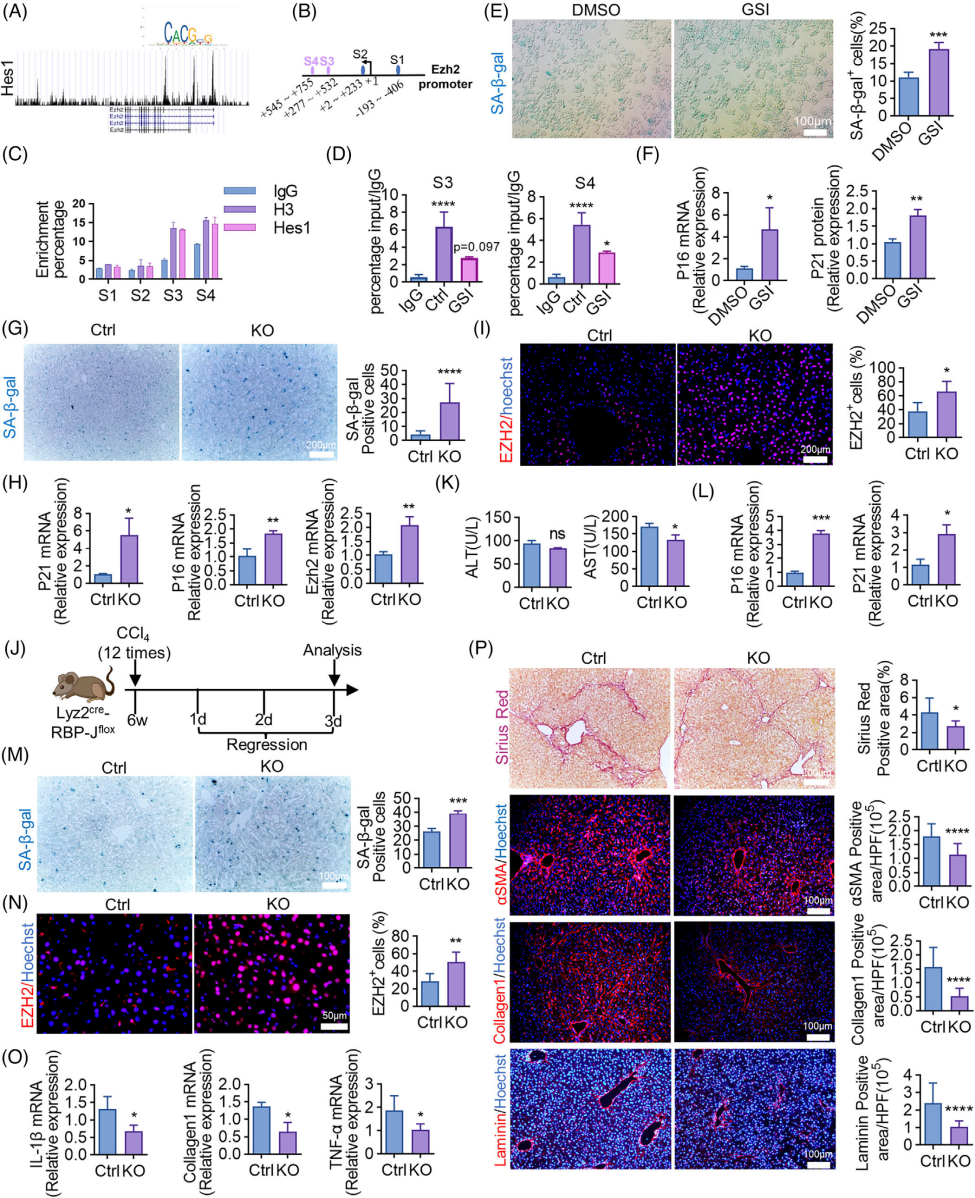
Notch‐Hes1 impedes fibrosis regression by suppressing the transcription of EZH2. (A) Hes1 binding motif logo (https://jaspar.genereg.net/analysis). The relationship between promoter region of EZH2 and Hes1 was shown graphically by Cistrome Data Browser (http://cistrome.org/db/#/). (B) According to JASPAR database, EZH2 promoter region contains potential Hes1 binding sites. (C) Relative enrichment of EZH2 promoter sequences in control (IgG), positive control (H3), and Hes1 ChIPs in RAW264.7 cells. (D) Relative enrichment of EZH2 promoter sequences in control (IgG) and Hes1 ChIPs in RAW264.7 cells treated with GSI. (E) RAW264.7 cells were cultured in vitro and stained by SA‐β‐gal. SA‐β‐gal‐positive cells were quantitatively compared between GSI‐treated and DMSO control groups. Scale bar: 100 μm. (F) Relative mRNA expressions of P16 and P21 were determined in RAW264.7 cells treated with GSI or DMSO. (G) Liver sections collected from KO (Lyz2^cre^‐RBP‐J^f/f^, homozygous) and control (Lyz2^cre^‐RBP‐J^f/+^, heterozygous) mice were stained by SA‐β‐gal. SA‐β‐gal‐positive cells were quantitatively compared. Scale bar: 200 μm. (H) Relative mRNA expressions of P16, P21, and EZH2 were determined in KO and control mice. (I) IF staining of EZH2. EZH2‐positive cells were quantitatively compared. Scale bar: 100 μm. (J) The strategy of regression established in KO and control mice. All the experimental mice were sacrificed and analyzed on regression day 3. (K) Serum ALT, AST levels in KO and control mice. (L) Relative mRNA expressions of P16 and P21 were determined in KO and control mice. (M) SA‐β‐gal staining of livers of KO and control mice. SA‐β‐gal‐positive cells were quantitatively compared. Scale bar: 100 μm. (N) IF staining and quantification of EZH2 in livers of KO and control mice. Scale bar: 50 μm. (O) Relative mRNA expressions of IL‐1β, Collagen1, TNF‐α were determined in KO and control mice. (P) Sirius red, αSMA, Collagen1, and Laminin staining of livers of KO and control mice. Positive staining areas were quantitatively compared. Scale bar: 100 μm (αSMA, Collagen1, Laminin), scale bar: 200 μm (Sirius red). Bars = means ± SD; *n* = 2–4; *p < 0.05, **p < 0.01, ***p < 0.001, ****p < 0.0001.

After analyzing the promoter region of EZH2, we discovered multiple putative binding sites for Hes1 transcription repressor (Figure 7A,B). ChIP (chromatin immunoprecipitation)‐qPCR results demonstrated a significant binding affinity of Hes1 to the site3 and site4 of EZH2 promoter. Blockade of Notch signaling using GSI (gamma‐secretase inhibitor)‐treated macrophages further significantly inhibited this enrichment efficiency (Figure 7C,D), suggesting Hes1 inhibits EZH2 transcription by interacting with the EZH2 promoter region.

We then observed the regulatory effect of Notch‐Hes1 signaling on cellular senescence. In the in vitro study, GSI, a classical Notch signaling inhibitor, significantly enhanced SA‐β‐gal^+^ stain (Figure 7E) and increased P16 and P21 mRNA levels (Figure 7F) in RAW264.7 macrophages. In the in vivo study, inactivation of macrophage‐derived Notch signaling was achieved by constructing Lyz2^Cre^‐RBP‐J^f/f^ transgenic mice as previously we did.^39^ Disruption of Notch/RBP‐J in macrophages, triggered hepatic cellular senescence (Figure 7G,H) and upregulated the mRNA level of EZH2 (Figure 7H). IF staining confirmed the increased EZH2 expression in livers of Lyz2^Cre^‐RBP‐J^f/f^ mice (Figure 7I). These data implied that macrophage‐derived Notch signaling deactivated senescence by impeding EZH2. During the process of fibrosis regression (Figure 7J), transgenic inactivation of Notch signaling decreased serum AST but not ALT level (Figure 7K). Meanwhile, aggravated hepatic cellular senescence, relieved fibrosis, and increased expression of EZH2 were detected in Lyz2^Cre^‐RBP‐J^f/f^ mice (Figure 7L–P), proving Notch blockage in macrophages facilitated senescence‐triggered fibrosis regression.

### 2.8 Blocking EZH2 abrogates the increased senescence and accelerated fibrosis regression caused by Notch deficiency

To testify whether EZH2 blockage could reverse Notch deficiency‐induced effects, EPZ6438 was adopted in Lyz2^Cre^‐RBP‐J^f/f^ and control mice according to the strategy shown in Figure S2A. As expected, EPZ6438 largely restored the enhanced senescence, but deteriorated liver function in Notch‐deficient mice (Figure S2B,C). Moreover, blocking EZH2 decelerated liver senescence and fibrosis regression in Lyz2^Cre^‐RBP‐J^f/f^ mice (Figure S2D–F), suggesting that Notch counteracted senescence‐driven fibrosis regression through regulating epigenetic EZH2 signaling.

### 2.9 Notch inhibitor augments senescent cells and promotes fibrosis regression

**FIGURE 8:**
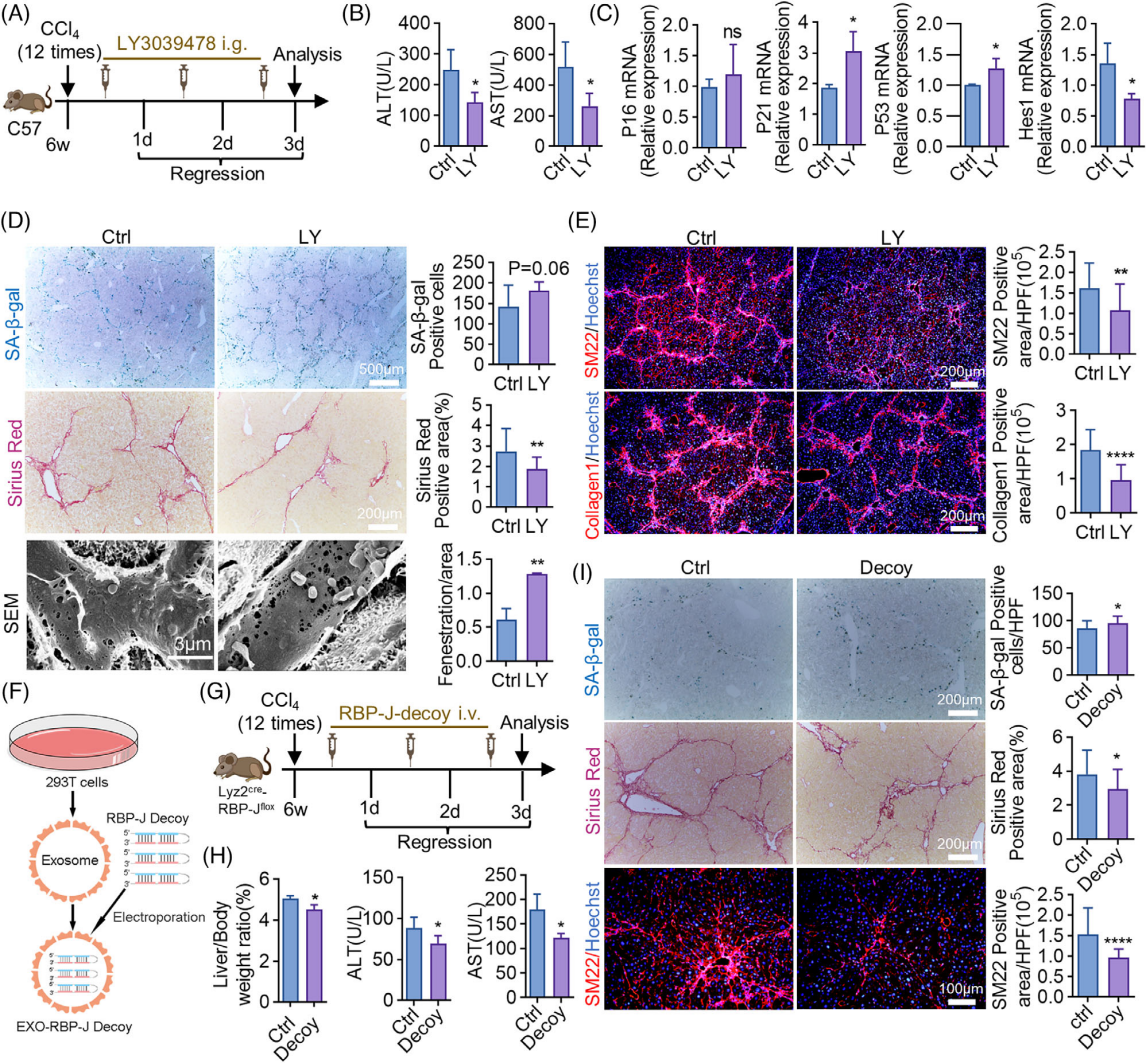
Blocking Notch signaling facilitates senescence‐induced fibrosis regression. (A) The strategy of LY3039478 administration. All the experimental mice were sacrificed and analyzed on regression day 3. (B) Serum levels of ALT and AST in LY3039478‐treated (LY) and control mice. (C) Relative mRNA levels of P16, P21, P53, and Hes1 were detected by RT‐qPCR in LY3039478‐treated or control mice. (D and E) Staining of SA‐β‐gal, Sirius red, SEM, Collagen1, and SM22 in mice subjected to LY3039478 treatment or not. Positive areas were quantified and compared. Scale bar: 500 μm (SA‐β‐gal), scale bar: 200 μm (Sirius red, SM22, Collagen1), scale bar: 3 μm (SEM). (F) Schematic representation of RBP‐J decoy loaded into HEK293T‐derived exosomes. (G) The strategy of the usage of exosome‐mediated RBP‐J decoy. All the experimental mice were sacrificed and analyzed on regression day 3. i.v. = intravenous injection. (H) Detection of liver/body weight, serum ALT, and AST in RBP‐J decoy‐treated (decoy) and control mice. (I) Staining of SA‐β‐gal, Sirius red, and SM22 in livers of RBP‐J decoy‐treated and control mice. SA‐β‐gal‐positive cells, Sirius red and SM22 positive areas were quantitatively compared. Scale bar: 200 μm (SA‐β‐gal, Sirius red), scale bar: 100 μm (SM22). Bars = means ± SD; *n* = 4–5; *p < 0.05, **p < 0.01, ****p < 0.0001; ns, not significant.

To confirm the effect of Notch, LY3039478, another Notch inhibitor was administrated during the process of regression (Figure 8A). LY3039478, which recovered liver malfunction (Figure 8B), upregulated the expression of senescence‐associated genes and inactivated Notch downstream gene Hes1 (Figure 8C). Blocking Notch by LY3039478 stimulated hepatic cellular senescence and accelerated fibrosis regression (Figure 8D,E).

Previously, our group developed an exosome‐mediated RBP‐J decoy oligodeoxynucleotides (ODNs) (Figure 8F), which could be delivered to hepatic macrophages to ameliorate CCl_4_‐induced fibrosis by abrogating Notch/RBP‐J signaling.^40^ Thus, exosome‐mediated RBP‐J decoy was then manipulated to explore the therapeutic role of Notch in fibrosis regression (Figure 8G). RBP‐J decoy reduced relative liver weight and serum ALT and AST levels (Figure 8H). To be noted, targeted delivery of exosome‐mediated RBP‐J decoy successfully stimulated senescence and fibrosis regression (Figure 8I). All these findings implied that targeting Notch signaling provided a potential way to promote fibrosis regression.

## 3 DISCUSSION

Liver fibrosis due to viral, metabolic, or alcoholic chronic liver diseases is caused by excessive accumulation of extracellular matrix.^41^ Aggravated liver fibrosis leads to cirrhosis, portal hypertension, liver carcinoma, and liver failure, making a big challenge to global health.^42^ Thus, understanding the involved mechanisms of fibrogenesis is of great importance. Once the causative effects of chronic liver diseases cease, established fibrosis is reversable, which is called regression.^43^ Growing studies have highlighted the importance of liver fibrosis regression,^5^ because it provides a promising therapeutic strategy to treat liver fibrosis.

In experimental investigations, the reversal of CCl_4_‐induced liver fibrosis could be detected in 1 or 2 weeks following the cessation of CCl_4_ toxicity, which was usually determined by evaluation of fibrotic markers.^44^ In this study, we observed that the regression of fibrosis initiated 3 days following the removal of CCl_4_, as desmin and Masson stain significantly decreased at that time. Meanwhile, senescent cells accumulated in the recovered liver. The coincidence that the initiation of regression and emergence of senescence appeared at the same time aroused our great interests. To testify our hypothesis that the augmentation of senescent cells is the determinant of fibrosis regression, senescent cells were removed by senolytics. Interestingly, clearance of senescent cells after CCl_4_ removal, deteriorated liver function and increased the expression of fibrotic markers, indicating that the regression of fibrosis was disturbed. Thus, we may conclude that the emerged senescent cells are beneficial to protect the injured liver against CCl_4_ toxicity by advancing fibrosis regression.

After confirming the beneficial effect of senescence, the origin of senescent cells should be determined. Previously, Krizhanovsky et al. used IHC staining to show that senescent cells, which limited fibrogenesis, were activated stellated cells.^15^ In our study, FCM first identified that liver NPCs, particularly macrophages and endothelial cells, constituted the major proportion of senescent cells. To confirm these findings, scRNA‐seq was applied in livers of mice with CCl_4_ removal. Although senescence‐associated genes enriched in macrophages, endothelial cells, and myofibroblasts, the biggest alteration of P16 and P21, two landmark genes of senescence, was speculated in macrophages. IF staining also confirmed the co‐stain of hepatic macrophages and senescence. In subsequent studies, either clearance of senescent cells or macrophages reduced hepatic senescent macrophages. Even if we could not conclude that senescent cells originated from macrophages, our findings illustrated that macrophages devoted great contribution to regression‐related senescence. Recently, Satotaka et al. confirmed the existence of a cluster of P16^high^ hepatic senescent cells, which shared the expression of both endothelium and macrophage markers in fibrotic liver by scRNA‐seq.^45^ Thus, it seems to be extremely complicated to identify the constitution of fibrosis‐associated senescent cells currently. Additionally, different injury models may lead to different cellular senescence during fibrosis. Our preliminary investigations suggested that non‐alcoholic steatohepatitis (NASH)‐associated fibrosis dominantly induced hepatocyte senescence; however, bile duct ligation‐induced fibrosis probably triggered senescence of biliary epithelial cells.

The decisive role of macrophages in promoting inflammatory response during fibrogenesis has been extensively discussed.^46^ In this study, a contradictory finding is that aggravated fibrosis was accompanied with decreased macrophages while senescent cells were removed. To elucidate this phenomenon, the polarization of macrophages was analyzed. Interestingly, the decrease of hepatic macrophages was due to the reduction of M2 macrophages, which mainly exerted anti‐inflammatory effect.^47^ Moreover, clearance of macrophages disrupted fibrosis regression as well, implying that depleted macrophages were predominantly anti‐inflammatory ones. Therefore, emerged senescent cells may initiate liver fibrosis regression through igniting the anti‐inflammatory attribute of macrophages.

As emerged senescence is beneficial to maintain fibrosis regression, stimulation of such senescence would be a protective way against liver fibrosis. In this study, the epigenetic modulator EZH2 was found to be transcriptionally suppressed by Notch‐Hes1 signaling. Inactivation of Notch in transgenic mice facilitated fibrosis regression by augmenting EZH2‐regulated senescence. For translational application, Ly3039478, a Notch inhibitor, was used to trigger senescence, which successfully accelerated fibrosis regression. However, the effect of Notch deficiency caused by Ly3039478 is global. Constructing a targeted delivery of Notch inhibitor to injured liver is an even better way to interfere fibrosis. RBP‐J is the core transcriptional factor of Notch signaling pathway.^48^ Previously, our group developed RBP‐J decoy ODNs, which inactivated Notch signaling by suppressing RBP‐J.^40^ We found that injected exosome‐equipped RBP‐J decoy was mainly endocytosed by hepatic macrophages,^40^ providing us with a promising strategy to target fibrotic liver. In this study, exosome‐mediated RBP‐J decoy promoted liver fibrosis regression by enhancing senescence. Thus, targeting Notch signaling provides an appealing strategy for the treatment of liver fibrosis.

Apart from regression, we also evaluated senescence during fibrosis progression. We found that senescent cells accumulated 1 week after CCl_4_ administration (Figure S3A). Once macrophages were depleted at the beginning of fibrosis progression (Figure S3B), senescence was diminished and fibrosis was reversed (Figure S3C). Besides, removing senescent cells by DQ also alleviated CCl_4_‐induced fibrosis (Figure S4A–F). These data suggested that senescent cells probably play distinct roles in regulating progression and regression of liver fibrosis. To confirm this phenomenon, additional work should be carried out in the future. Besides, some other limitations are involved into this study. Precise identification of the origin of senescent cells should be considered in future work. Linage tracing using P16 or P21‐cre mice may provide a better way to distinguish which hepatic cells conducted senescence during regression. Although we presumably thought that senescent cells exerted M2 macrophage‐like anti‐inflammatory effects during regression, whether the polarization of hepatic macrophages changed after EZH2 or Notch disruption was not investigated in the current work. All these concerns need to be clarified in subsequent studies.

In conclusion, our study first unraveled that liver fibrosis regression was driven by macrophage senescence. Once anti‐inflammatory senescent macrophages were abolished, fibrosis regression was terminated. Mechanistically, EZH2, which was transcriptionally impeded by Notch, strictly governed senescence‐induced regression. Blocking Notch may treat fibrosis by augmenting EZH2‐regulated senescence.

## 4 MATERIALS AND METHODS

### 4.1 Reagents

Dasatinib (SC0150) was purchased from Beyotime. Quercetin (S2391), DAPT (S2215), and LY3039478 (S7169) were purchased from Selleck. EPZ6438 (T1788) was purchased from Targetmol. Clodronate liposomes (40337ES10) were procured from Yeasen. The antibodies information used in the article is listed in Table S1.

### 4.2 Mice

Male C57BL/6J mice (8 weeks old) were purchased from Charles River Laboratories. Mice of the C57BL/6 genetic background were bred in specific pathogen‐free (SPF) facility. Lyz2‐Cre mice (Jackson Laboratory, 004781) were crossed with RBP‐J‐flox mice.^39^ Genomic DNA from the mouse tail was used as a template for PCR analysis of offspring genotypes.

To induce liver fibrosis, mice were intraperitoneally injected with olive oil or CCl_4_ (0.6 μL/g, 15% with olive oil) for 6 weeks.^49^ The regression of fibrosis was evaluated 3 days following the last injection of CCl_4_. During the process of liver fibrosis, DQ (D: 5 mg/kg, Q: 50 mg/kg),^50^ clodronate liposomes (50 mg/kg),^51^ LY3039478 (10 mg/kg),^52^ and EPZ6438 (34 mg/kg)^53^ were used for specific experiments.

All animal experiments were performed in accordance with the principles approved by the Animal Experiment Administration Committee of the Fourth Military Medical University and with humanitarian care.

### 4.3 Single‐cell sequencing analysis

Single‐cell RNA sequencing was performed with the assistance of Guangzhou Kidio Biotechnology, as we previously described.^49^ GSVA analyses were carried out according to the published literature.^54^

### 4.4 Cell isolation

Isolation of mouse liver nonparenchymal cells was performed according to a two‐step perfusion procedure described previously.^55^ Anti‐F4/80 and anti‐CD146 magnetic beads were used for Kupffer cell and LSEC isolation, respectively.

### 4.5 Fluorescent microsphere assay

Blood flow alterations were determined by injection of 15 μm fluorescent polystyrene microspheres (100 μL, F8842, Invitrogen) into the spleen. Determination of fluorescent microspheres was performed as described previously.^19^

### 4.6 Chromatin immunoprecipitation assay

Chromatin immunoprecipitation assays were performed using the SimpleChIP Enzymatic Chromatin IP kit (CST, 9003) and anti‐Hes1 antibody (CST, 11988) according to the manufacturer's instructions.^19^

### 4.7 Exosomes loaded with RBP‐J decoy oligodeoxynucleotides

#### 4.7.1 Extraction of exosomes from 293T cells

293T cells were overgrown with adherents, and the supernatant was collected after 48 h of culture with serum‐free medium. The cell supernatant was centrifuged: 500 × *g*/5 min, 3000 × *g*/30 min, and the supernatant was incubated with PEG6000 overnight. The next day, the supernatant incubated overnight with PEG6000 was centrifuged at 12,000 × *g*/1 h. The supernatant was discarded, and the precipitate (exosomes) was resuspended with an appropriate amount of sterile PBS.

#### 4.7.2 Electroporation

The nucleic acid sequence of the RBP‐J decoy ODNs (decoy RBP‐J) was 5′‐CTGCGTGGGAACTAGCGTGGGAATATTTTTTATATTCCCACGCTAGTTCCCACGCAG‐3′; the nucleic acid sequence of the control decoy ODNs (decoy Ctrl) was 5′‐CTGCGTTTTAACTAGCGTTTTAATATTTTTTATATTAAAACGCTAGTTAAAACGCAG‐3′.^40^ Two RBP‐J binding sites (CGTGGGAA) were present in the decoy, with three phosphorothioate‐modified sites at each end.^40^ All the ODNs were synthesized exclusively by Tsingke Biotechnology Co. (Beijing, China). Each mouse was mixed with RBPJ‐Decoy/control decoy ODNs (2.5 nmol) and exosomes (BCA quantification of 200 μg) in a 0.4 cm electric piercing cup (Bio‐Rad), supplemented with a certain amount of electric piercing buffer (Bio‐Rad). electroporated with 400 V and 125 μF. Rewarming in the incubator at 37°C for 30 min. After mixing the liquid in the electric perforator cup with one‐third volume of PEG6000, centrifugation performed at 12,000 × *g*/15 min. The discarded supernatant was resuspended with sterile PBS.

#### 4.7.3 Exosome injection

Exosomes containing RBPJ/control decoy ODNs were administered intravenously via the tail vein for three times per 3 days when 3 days after ablation in liver fibrosis model. Mice were euthanased after 3 days of CCl_4_ withdrawal for further experiments.

### 4.8 Senescence‐associated β‐galactosidase staining

Senescence was assessed by staining 8–10 μm frozen sections of mouse liver^19^ using Senescence β‐Galactosidase Staining Kit (Beyotime, C0602), according to the manufacturer's instructions.

### 4.9 Flow cytometry

The flow cytometry analysis was conducted in accordance with the previously established protocol.^56^ An overview of antibody information can be found in Table S1.

### 4.10 Apoptosis assay

Cell apoptosis was assessed via TUNEL assay utilizing the One Step TUNEL Apoptosis Assay Kit (Beyotime, C1089). Refer to the manufacturer's guidelines for comprehensive staining protocols.

### 4.11 Scanning electron microscopy

The scanning electron microscopy was conducted in accordance with the previously established protocol.^48^

### 4.12 Real‐time quantitative PCR assay

Total RNA from primary macrophages or liver tissues was harvested in Trizol reagent. Evo M‐MLV RT Kit (Accurate Biology, AG11728) was employed to synthesize cDNA. The SYBR Green PCR Master Mix (Accurate Biology, AG11701) was utilized for performing RT‐qPCR analysis using the Bio Rad C1000 Touch instrument. Actin was utilized to normalize the RT‐qPCR data. The primer pairs used in this article are listed in Table S2.

### 4.13 Western blot analysis

Liver tissues or macrophages were lysed with RAPI. Subsequently, the mixture was treated with grinding plus ultrasonic crushing. After being centrifuged, the supernatant was removed and incubated for 10 min at 100°C with 5× SDS. The BCA protein quantification kit (Thermo Fisher Scientific) was used to determine protein content. SDS–polyacrylamide gel electrophoresis was used to separate equal amounts of protein, which was then transferred to PVDF membranes. After 5% milk blocking, the primary antibody and corresponding secondary antibody were incubated (antibody information refers to Table S1), and finally the protein band was exposed with an instrument The protein signals were captured by the Chemi Doc XRS+ System (Bio‐Rad Laboratories, Inc.), followed by the analyses using the ImageJ software.

### 4.14 Histological staining

The perfused liver was soaked in 4% paraformaldehyde for 4–12 h. A frozen section was made by dehydrating tissue in 30% sucrose overnight, embedding it in OCT, and sectioning it at 8−10 μm. Immunofluorescence (IF) and immunohistochemistry (IHC) staining were completed as previously described conducted.^55^ The statistical methods for the images of IHC were the number of α‐SMA, Sirius Red and Collagen1‐positive pixels (SUM value output in the software)/the resolution of the Image (2048 × 1536) ×100%. An overview of antibody information can be found in Table S1.

### 4.15 Statistical analysis

GraphPad Prism was used for statistical analysis, and data were reported as mean  ±  SD. An unpaired *t*‐test (Student's *t*‐test) was used to make group comparisons. *p*‐Values < 0.05 were considered as a measure of statistical significance. *p < 0.05, **p < 0.01, ***p < 0.001, ****p < 0.0001; ns, not significant.

## AUTHOR CONTRIBUTORS

PS, JLD, and JD conducted the experiments. JJL, ZQF, HX, ZWL, and WD provided assistance in animal experimentation. MX, YWL, and FH aided in data collection. KST and LW developed the experimental design. LW authored the article. All authors have read and approved the final manuscript.

## CONFLICT OF INTEREST STATEMENT

The authors declare that they have no conflicts of interest.

## ETHICS APPROVAL

The Animal Experiment Administration Committee of the Fourth Military Medical University reviewed and approved all animal experiments to ensure ethical and humane treatment of animals (KY20223331‐1).

## Supporting information

Supporting Information

## Data Availability

The data utilized in this study can be made available upon request from the corresponding author.
