# Supplementary material for: Cellular senescence primes liver fibrosis regression through Notch‐EZH2
Source: MedComm (2020). 2023 Aug 21;4(5):e346. doi: 10.1002/mco2.346 (PMC10442476; doi:10.1002/mco2.346)
Supplement: Supplementary file 1 — Supporting Information [file MCO2-4-e346-s001.docx]

**Supplementary data**

**Cellular senescence primes liver fibrosis regression through Notch-EZH2**

Ping Song^1,#^ Juan-Li Duan^1,#^ Jian Ding^1,#^ Jing-Jing Liu^1^ Zhi-Qiang Fang^1^ Hao Xu^1^ Zhi-Wen Li^1^ Wei Du^1^ Ming Xu^1^ Yu-Wei Ling^1^ Fei He^1^ Kai-Shan Tao^1,*^ Lin Wang^1,*^

^1^Department of Hepatobiliary Surgery, Xi-Jing Hospital, Fourth Military Medical University, Xi’an 710032, China

^#^ These authors contributed equally to this study.

^*^ **Correspondence to:** Lin Wang, Department of Hepatobiliary Surgery, Xi-Jing Hospital, Fourth Military Medical University, Xi’an 710032, China, Email: [fierywang@163.com](mailto:fierywang@163.com); Kai-Shan Tao, Department of Hepatobiliary Surgery, Xi-Jing Hospital, Fourth Military Medical University, Xi'an 710032, China, Email: [taokaishan0686@163.com](mailto:taokaishan0686@163.com).


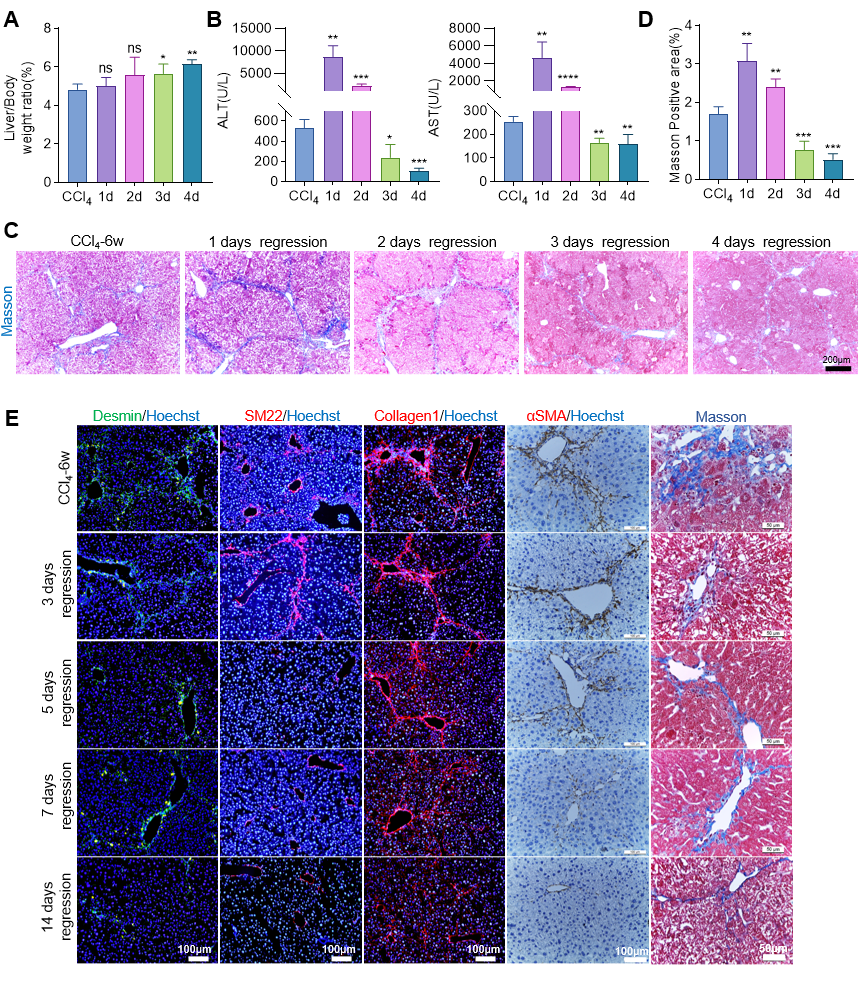


**Fig.S1 The evaluation of liver fibrosis regression.**

(A) Liver weight/body weight ratio at different time points after CCl_4_-withdrawal. (B) Serum levels of ALT, AST at different time points of liver fibrosis regression. (C) Masson staining at different time points of regression. Positive areas were quantified and compared (D). Scale bar: 200 μm. (E) IF (Desmin, SM22, Collagen1) and IHC (αSMA, Masson) staining of livers on day 3, day 5, day 7 and day 14 of regression. Mice sacrificed immediately after the 12th CCl_4_ injection served as control. Positive areas were quantified and compared. Scale bar: 100 μm (Desmin, SM22, Collagen1, αSMA), scale bar: 50 μm (Masson). Bars=means ± SD; *P < 0.05, **P < 0.01, ***P < 0.001, ns, not significant.


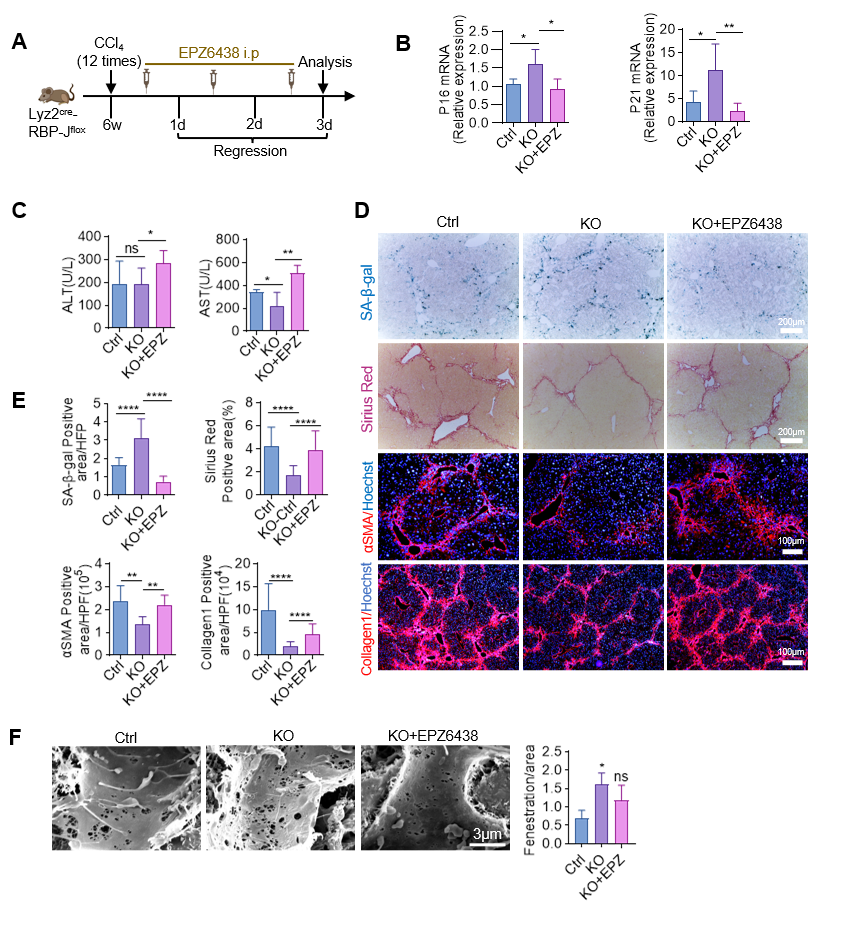


**Fig.S2 Blocking EZH2 in Notch deficient mice.**

(A) The strategy of EPZ6438 administration in KO (Lyz2^cre^-RBP-J^f/f^) and control (Lyz2^cre^-RBP-J^f/+^) mice. (B) RT-qPCR analyses of P16 and P21 in control, KO and KO+EPZ6438 group. (C) Detection of serum ALT, AST in control, KO and KO+EPZ6438 group. (D) SA-β-gal, Sirius red, αSMA and Collagen1 staining of livers of control, KO and KO+EPZ6438 mice. Scale bar: 200 μm (SA-β-gal, Sirius Red), scale bar: 100 μm (αSMA, Collagen1). (E) Quantification of (D). Positive staining areas were quantitatively compared. (F) SEM staining of livers collected from control, KO and KO+EPZ6438 mice. Scale bar: 3 μm. Bars=means ± SD; *P < 0.05, **P < 0.01, ****P < 0.0001, ns, not significant.


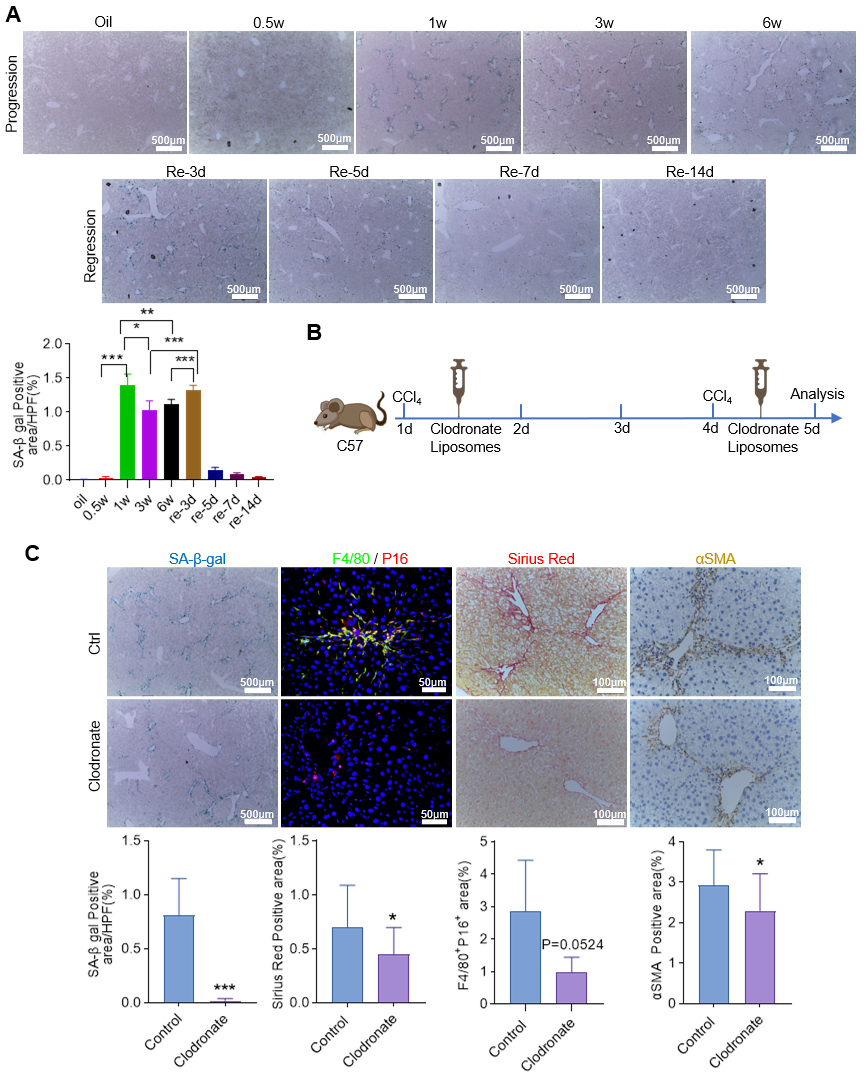


**Fig.S3 Clearance of macrophages during the progression of liver fibrosis.**

(A) Frozen sections of livers collected from different time points of CCl_4_-induced fibrosis progression and regression were stained with SA-β-gal. Positive areas of SA-β-gal were quantitatively compared. Scale bar: 500 μm. (B) The strategy of clodronate liposome injection. Clodronate liposomes were injected at day 1.5 and 4.5. All the experimental mice were sacrificed and analyzed on the 5th day after CCl_4_ injection. (C) Frozen sections of fibrotic livers were stained with SA-β-gal Kit, F4/80 (green) and P16 (red). Paraformaldehyde fixed liver sections were stained with Sirius Red and αSMA to assess the degree of fibrosis. Positive areas were quantitatively compared. Scale bar for SA-β-gal staining: 500 μm. Scale bar for immunohistochemical staining :100 μm. Scale bar for immunofluorescence staining :50 μm. Bars = means ± SD; n = 4; *P < 0.05, **P < 0.01, ***P < 0.001, ns, not significant.


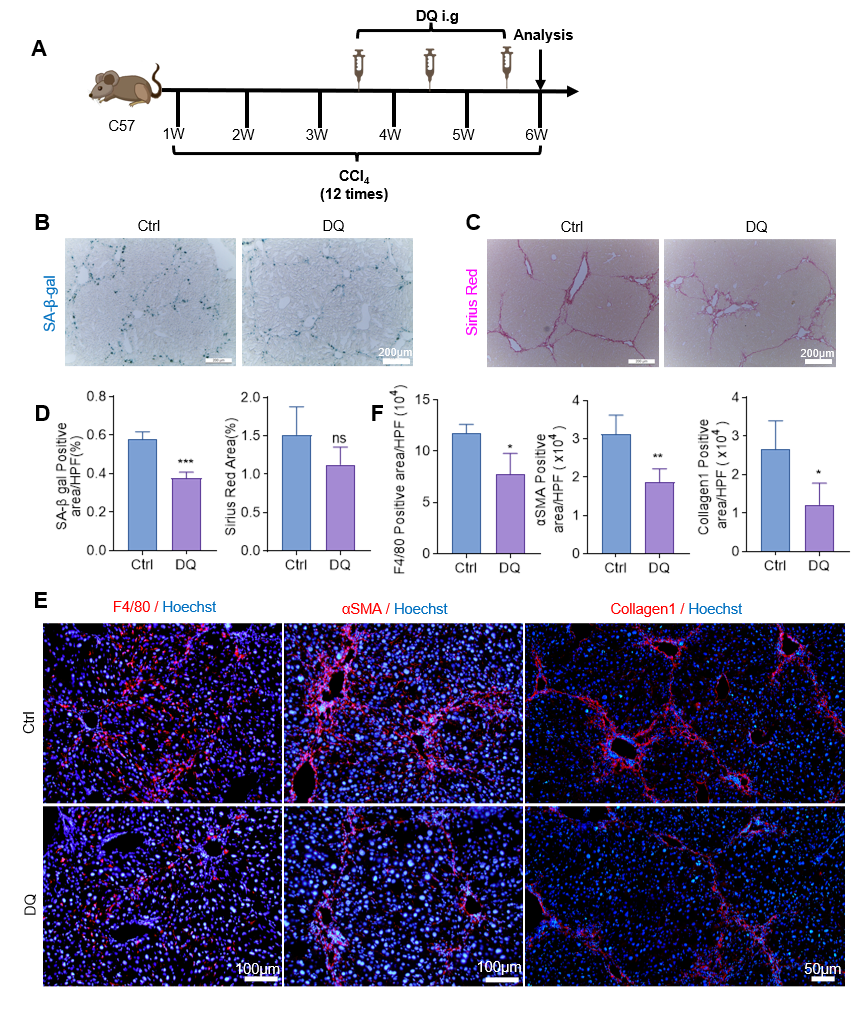


**Fig.S4 Removal of senescent cells during the progression of liver fibrosis**

(A) The strategy of DQ administration in CCl_4_-induced fibrosis models. (B) Liver sections collected from DQ-treated and control mice were stained by SA-β-gal staining kit. Scale bar: 200 μm. (C) Sirius red staining of liver sections from DQ-treated or control mice. Scale bar: 200 μm. (D) SA-β-gal positive cells and positive areas of Sirius red were quantified and compared. (E) IF staining of F4/80, αSMA, Collagen1 in DQ-treated and control mice. Scale bar: 100 μm (F4/80, αSMA), scale bar: 50 μm (Collagen1). (F) The quantification of positive areas shown in (E). Bars=means ± SD; *P < 0.05, **P < 0.01, ***P < 0.001, ns, not significant.

**Supplementary Table1.**Antibodies used in this study

| Antibody | Supplier | Cat No. | Purpose |
| --- | --- | --- | --- |
| Lyve-1 | Reliatech | 103-M130 | IF |
| F4/80 | eBioscience | 14-4801082 | IF |
| α-sma | Abcam | ab124964 | IF |
| P16 | Abcam | ab51243 | IF |
| Desmin | Abcam | ab32362 | IF |
| Sm22 | Abcam | ab14106 | IF |
| Collagen1 | Abcam | ab34710 | IF |
| Laminin | Abcam | Ab7463 | IF |
| γ-H2AX | Servicebio | GB111841 | IF |
| EZH2 | CST | 5246 | IF/WB |
| Goat anti-Rabbit FITC | JacksonImmunoResearch | 111-095-144 | IF |
| Goat anti-Rabbit Cy3 | JacksonImmunoResearch | 111-165-003 | IF |
| Donkey anti-Rat Alexafluo594 | JacksonImmunoResearch | A21209 | IF |
| Donkey anti Rat fluo488 | Invitrogen | A21208 | IF |
| F4/80 | Servicebio | GB113373 | IHC |
| α-sma | Servicebio | GB111364 | IHC |
| P21 | ABclonal | A1483 | WB |
| P53 | ABclonal | A10610 | WB |
| P16 | Proteintech | 10883-1-AP | WB |
| Cleaved Caspase-3 | Abcam | Ab214430 | WB |
| Bax | CST | 5023S | WB |
| GAPDH | CST | 5174 | WB |
| Goat anti-Mouse-HRP | Abbkine | A21010 | WB |
| Goat anti-Rabbit-HRP | Abbkine | A21020 | WB |
| F4/80-PE | Biolegend | 123109 | FACS |
| CD11b-APC | eBioscience | 17-0112-81 | FACS |
| VEGFR2-APC | Biolegend | 136405 | FACS |
| Desmin-PE | Abcam | Ab224935 | FACS |
| Ly6C-PE | Biolegend | 128007 | FACS |
| F4/80-488 | Biolegend | 123119 | FACS |
| APC-CD11b | eBioscience | 17-0112-81 | FACS |
| CD80-FITC | Biolegend | 104715 | FACS |
| CD206-FITC | Biolegend | 141703 | FACS |
| Hes1 | CST | 11988 | CHIP |
| H3 | CST | 4620 | CHIP |
| IgG | CST | 2729 | CHIP |

**Supplementary Table2.** Primers used for qPCR

| **qPCR** | **Forward(5’-3’)** | **Reverse(5’-3’)** |
| --- | --- | --- |
| CDKN2a | GCCCAACGCCCCGAACTCTTTC | GCGACGTTCCCAGCGGTACACA |
| CDKN1a | CCTGGTGATGTCCGACCTG | CCATGAGCGCATCGCAATC |
| P53 | GCGTAAACGCTTCGAGATGTT | TTTTTATGGCGGGAAGTAGACTG |
| EZH2 | AGTGACTTGGATTTTCCAGCAC | AATTCTGTTGTAAGGGCGACC |
| Arg-1 | AGACAGCAGAGGAGGTGAAGAG | CGAAGCAAGCCAAGGTTAAAGC |
| Ym-1 | CATTCAGTCAGTTATCAGATTCC | AGTGAGTAGCAGCCTTGG |
| IL-10 | CCCTTTGCTATGGTGTCCTT | TGGTTTCTCTTCCCAAGACC |
| MR | AAACACAGACTGACCCTTCCC | GTTAGTGTACCGCACCCTCC |
| TNF-α | CTGAACTTCGGGGTGATCGG | GGCTTGTCACTCGAATTTTGAGA |
| Collagen1 | GCTCCTCTTAGGGGCCACT | ATTGGGGACCCTTAGGCCAT |
| MMP13 | TGTTTGCAGAGCACTACTTGAA | CAGTCACCTCTAAGCCAAAGAAA |
| Sm22 | CCAACAAGGGTCCATCCTACG | ATCTGGGCGGCCTACATCA |
| α-sma | CCCAGACATCAGGGAGTAATGG | TCTATCGGATACTTCAGCGTCA |
| IL-1β | GCAACTGTTCCTGAACTCAACT | ATCTTTTGGGGTCCGTCAACT |
| β-actin | GGCTGTATTCCCCTCCATCG | CCAGTTGGTAACAATGCCATGT |
| EZH2-site1 | AAAGGAACCGCACTAACTTTATCTG | CTGAGCCAAGTTTGAAATAGTTCAA |
| EZH2-site2 | CCCGAGAACCACTCAGCG | ACCACAGTCGCTGTCTTTGTTCTTT |
| EZH2-site3 | GCAAAAGAACAAAGACAGCGACT | CAATCGCCATCGCTTTTATTT |
| EZH2-site4 | TGGGACATCGAAGGCAGTGGA | AAAAGCGGCGCTCGAGTGAGAC |
| EZH2-site5 | GAGGGCTAGGGGACAGCTTTCT | ACACAAAGAAGACTTGAGGCTCAGC |
| Lyz2-cre | CCGGTCGATGCAACGAGTGATGAGG | GCCTCCAGCTTGCATGATCTCCGG |
| RBP-J-WT | GTTCTTAACCTGTTGGTCGGAACC | GCTTGAGGCTTGATGTTCTGTATTGC |
| RBP-J-mutant | GTTCTTAACCTGTTGGTCGGAACC | ACCGGTGGATGTGGAATGTGT |
